# Effects of the Combined Treatment with a G-Quadruplex-Stabilizing Ligand and Photon Beams on Glioblastoma Stem-like Cells: A Magnetic Resonance Study

**DOI:** 10.3390/ijms222312709

**Published:** 2021-11-24

**Authors:** Alessandra Palma, Sveva Grande, Anna Maria Luciani, Lucia Ricci-Vitiani, Mariachiara Buccarelli, Roberto Pallini, Alice Triveri, Valentina Pirota, Filippo Doria, Quintino Giorgio D’Alessandris, Francesco Berardinelli, Antonio Antoccia, Antonella Rosi

**Affiliations:** 1 National Centre for Innovative Technologies in Public Health, Istituto Superiore di Sanità, 00161 Rome, Italy; alessandra.palma@iss.it (A.P.); sveva.grande@iss.it (S.G.); annamaria.luciani@iss.it (A.M.L.); 2 Department of Oncology and Molecular Medicine, Istituto Superiore di Sanità, 00161 Rome, Italy; lucia.riccivitiani@iss.it (L.R.-V.); mariachiara.buccarelli@iss.it (M.B.); 3 Institute of Neurosurgery, Fondazione IRCCS Policlinico Universitario A. Gemelli, 00168 Rome, Italy; Roberto.Pallini@unicatt.it (R.P.); giorgiodal@hotmail.it (Q.G.D.); 4 Institutes of Neurosurgery, Università Cattolica del Sacro Cuore, 00168 Rome, Italy; 5 Department of Chemistry, University of Pavia, 27100 Pavia, Italy; alice.triveri01@universitadipavia.it (A.T.); valentina.pirota@unipv.it (V.P.); filippo.doria@unipv.it (F.D.); 6 Department of Science, University of Rome “Roma Tre” and INFN Sezione di Roma Tre, 00146 Rome, Italy; francesco.berardinelli@uniroma3.it (F.B.); antonio.antoccia@uniroma3.it (A.A.); 7 Laboratory of Neurodevelopment, Neurogenetics and Molecular Neurobiology, IRCCS Santa Lucia Foundation, 00143 Rome, Italy

**Keywords:** glioblastoma, stem cells, metabolism, magnetic resonance spectroscopy, photon beams, G4-quadruplex ligand

## Abstract

Glioblastoma multiforme is a malignant primary brain tumor with a poor prognosis and high rates of chemo-radiotherapy failure, mainly due to a small cell fraction with stem-like properties (GSCs). The mechanisms underlying GSC response to radiation need to be elucidated to enhance sensitivity to treatments and to develop new therapeutic strategies. In a previous study, two GSC lines, named line #1 and line #83, responded differently to carbon ions and photon beams, with the differences likely attributable to their own different metabolic fingerprint rather than to radiation type. Data from the literature showed the capability of RHPS4, a G-quadruplex stabilizing ligand, to sensitize the glioblastoma radioresistant U251MG cells to X-rays. The combined metabolic effect of ligand #190, a new RHPS4-derivative showing reduced cardiotoxicity, and a photon beam has been monitored by magnetic resonance (MR) spectroscopy for the two GSC lines, #1 and #83, to reveal whether a synergistic response occurs. MR spectra from both lines were affected by single and combined treatments, but the variations of the analysed metabolites were statistically significant mainly in line #1, without synergistic effects due to combination. The multivariate analysis of ten metabolites shows a separation between control and treated samples in line #1 regardless of treatment type, while separation was not detected in line #83.

## 1. Introduction

Glioblastoma (GBM) is the most lethal primary brain tumor and is associated with a median overall survival of 14 months. It inevitably recurs, even after surgical resection followed by intensive chemo-radiation therapy [1]. Recent studies have shown that glioblastoma stem cells (GSCs), a small fraction of self-renewing cells with stem-like properties, are responsible for tumor resistance to radiation and chemotherapy, as well as the stemness, quiescence, and therapy resistance that are maintained by GSC niches in the tumor microenvironment [2,3,4]. To enhance sensitivity to radiotherapy (RT), the mechanisms underlying the different cell responses to radiation need to be further elucidated and new strategies developed [5,6]. In fact, because of the high resistance to photon beam irradiation [7], the response of GSCs to irradiation with proton beams and charged particles has been examined [8,9]. However, results from these clinical trials (Cinderella and Cleopatra) conducted through a comparative analysis between protons, carbon ions and photon beams, are still pending [8,9]. Furthermore, using a 3D model for investigating GSC radiosensitivity to proton beam and carbon ion irradiation, Chiblack showed the enhanced biological effectiveness of carbon ion RT “in vivo” owing to its potent antiangiogenic effects and the eradication of radioresistant hypoxic tumor cells [10,11]. On the contrary, in a previous study, we demonstrated that the two patient-derived GSC lines #1 and #83, analysed by MRS and representative of different GSC metabolic and genetic profiles, showed a different metabolic response after treatment with photons and carbon ions [12]. The prevailing hypothesis was that the different response to radiation was attributable to genetic and metabolic differences between the two lines, representative of mesenchymal and proneural profiles, regardless of radiation type [12]. This behaviour can be attributed to the high heterogeneity of GSCs in primary tumors. In fact, like other cancers, GBM displays high heterogeneity among patients, with relevant differences in genomic, transcriptomic, proteomic, and metabolomic features, and quite different cell populations seem to be present in the same tumor [13,14]. Furthermore, subtypes of high-grade glioma have been identified based on molecular gene expression [15], which includes the proneural, proliferative, and mesenchymal subtypes. The hypothesis that the ability to escape or to mitigate radiation damage could be due to different subtype characteristics deserves to be further investigated. Both inter- and intra-individual heterogeneity may then be responsible for treatment failure and tumor relapse, suggesting the necessity of tailored therapies. GSC heterogeneity, in particular, should be targeted with different or combined approaches to overcome GSCs’ resistance to therapeutical treatments. Over the last 10 years, new preclinical experimental evidence has emerged, supporting the rationale of employing G-quadruplex (G4) ligands either as a single agent or in combination with ionizing radiation in the treatment of gliomas [16,17,18,19,20,21,22,23]. G4s are non-canonical nucleic acid base pairing structures found in G-rich regions of the human genome, such as telomeres, gene promoters, 5′ untranslated regions, and replication origins [24,25]. Targeting of G4 secondary structures has been proposed by employing a variety of small molecules showing G4-stabilizing properties with the aim of developing a new class of cancer drugs and, to date, the clinical applicability of G4s as anticancer drugs is currently being evaluated [24,26,27].

In a previous paper [22], it has been reported that the combination of photon beam irradiation and exposure to the G4-ligand RHPS4 induced a synergistic response on U251MG glioblastoma cells with respect to telomere dysfunction. Further studies conducted on the same cell model combining carbon ions and the G4-stabilising agent RHPS4 confirmed this hypothesis [28]. Again, DNA damage has been observed as a synergistic effect of RHPS4 and radiation regardless of radiation quality. These interesting results showed that telomeric interaction with G4-ligands induces telomere dysfunction and sensitizes U251MG glioblastoma cells to IR by increasing the frequency and complexity of chromosomal exchanges [22,28]. For this reason, drugs, including G4-ligands, affecting telomere structure and/or telomerase activity are currently seen as an attractive tool in oncology [28,29]. Unfortunately, RHPS4 recently showed severe cardiotoxicity [30], which precluded its use in clinics. Chemical analogues of RHPS4 have been investigated, with testing for their efficacy and reduced toxicity [30]. The modifications of the prototype RHPS4 allowed the synthesis and the selection of novel promising G4-stabilizing telomere targeting agents, these being superior to the standard acridinium salt both in terms of toxicological profile and on-target properties, which could be suitable compounds for progression into clinical trials [30]. Among these we decided to focus our studies on the most promising derivative, reported as Ligand #190 (compound **8**).

To gain greater insight into the synergistic effects of radiation and G4-ligands, we performed a metabolomic analysis with MR spectroscopy (MRS) to study changes occurring after GSC treatment with one of the RHPS4 analogues, namely, Ligand #190, and photon beam irradiation. Improved knowledge of the metabolism of brain tumors after chemo-radiotherapeutic treatment may contribute to the discovery of new diagnostic or prognostic biomarkers. In particular, metabolic reprogramming is a hallmark of GSCs [31] used to identify malignant signatures and open new ways to develop tailored therapeutic approaches. Stratifying patients according to molecular biomarker profiles is a key step in managing patient heterogeneity.

GSC lines #1 and #83 have been selected from a panel of patient-derived GSCs on the basis of their biological, metabolic, and genetic characteristics [7,12,32,33,34]; their response to photon beams and carbon ions is mainly due to their intrinsic characteristics rather than the quality of radiation treatments, as shown in a previous work [12]. These two lines are, then, good candidates to study the effect of the newly synthetized sensitizer’s ligand #190.

The synergistic effect of radiation and ligand #190 has been tested through MRS experiments at different times after ligand treatment and photon irradiation at 20 Gy; three different treatment conditions (only radiation, only ligand #190 treatment, combined radiation and ligand #190 treatment) were compared with the controls. Cell survival in these different conditions was also evaluated.

## 2. Results

**Figure 1:**
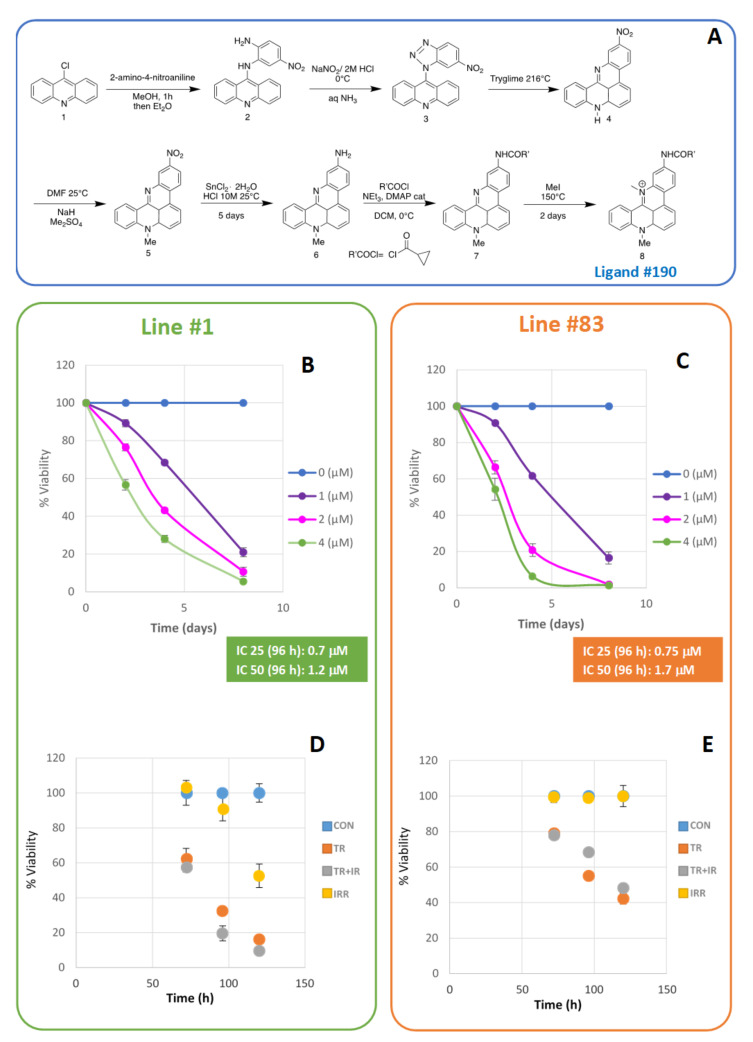
Synthetic protocols followed for the synthesis of target compound **8**, named ligand #190 (**A**). Dose-response curves for line #1 (**B**) and #83 (**C**) after treatment with ligand #190 as a function of time. The values of the extrapolated inhibition concentrations 25% (IC25) and 50% (IC50) at 96 h after treatment are indicated. Line #1 (**D**) and line #83 (**E**) percentage of viability shown as a function of time in the four different conditions (control—CON; ligand #190 treated—TR; photon beam irradiated—IR; and irradiated plus treated—IR + TR).

The target compound **8**, namely, ligand #190, was prepared according to the synthetic protocols outlined in Figure 1A. We introduced some changes to the published procedures used for the synthesis of compounds **2**–**6** to improve the global yields of the single steps, as described in the Supplementary Materials.

To obtain compound **8**, we performed a primary ammine acylation of **6** and subsequent methylation of **7**, following a known protocol not already reported in the literature, on our substrate. In brief, 2-amino-4-nitroaniline is first subjected to a nucleophilic substitution conducted in methanol treated with 9-chloroacridine 1 over 30 min. The mixture was refluxed for 90 min, cooled, and the product was precipitated with diethyl ether. Product 3 was obtained after a nitrosation of **2** with NaNO_2_ in HCl, and it was subsequently thermalized in Triglyme, reaching compound **4**. The methylation of **4** with NaH and dimethyl sulfate yielded 8-methyl-2-nitroquinoacridine **5**, which was then reduced to the corresponding amine **6** with stannous chloride in concentrated hydrochloric acid, after which the mixture was stirred at room temperature for 5 days. Then, to obtain the final product, we performed an acylation of **6** with the cyclopropanecarbonyl chloride, gaining the amide **7**, which was then heated with an excess of methyl iodide for 4 days at 150 °C in a pressure tube to obtain **8** (ligand #190) with a high yield (see Supplementary Materials and Figure S1–S3).

Two GSCs, namely, line #1 and #83, were selected from a panel of patient-derived GSCs on the basis of their characteristics; patient demographics and features are reported in Supplementary Table S1.

Dose-response curves for lines #1 and #83 after treatment with ligand #190 are reported in Figure 1B,C respectively. The ligand #190 inhibition concentrations of 25% (IC 25) and 50% (IC 50) were determined and the IC 25 value was identified for each line and used for the experiments (Figure 1B,C). Cell growth as a function of time in the four different conditions (untreated control cells—CON; ligand #190 treated cells—TR; photon beam irradiated cells—IR; and irradiated plus ligand #190 treated cells—IR + TR) is shown in Figure 1D,E (lines #1 and #83, respectively).

Cells of both lines in the four different conditions were then studied by MR spectroscopy. For combined treatment ligand plus irradiation experiments, cells were irradiated at a dose value of 20 Gy at day three after ligand 190 treatment and analyzed by 1D and 2D MR spectroscopy 24 and 48 h after irradiation (Supplementary Figure S4). Parallel MR experiments on control, ligand 190 treated, and irradiated cell samples were performed.

Ten metabolic signals were identified in 1D and 2D COSY spectra, and quantified in 2D. Assignments are reported in Supplementary Figure S5A,B.

Mobile lipid (ML) signals at 1.28 ppm and 0.89 ppm from the spectra of both cell lines are affected by the treatments, even if to differing extents. An increase of 2D A peak intensity (from mobile lipids CH_2_ n and terminal CH_3_ correlation) was observed late after the different treatments in the spectra of line #1, while this signal intensity showed a fluctuant behaviour in spectra of line #83.

**Figure 2:**
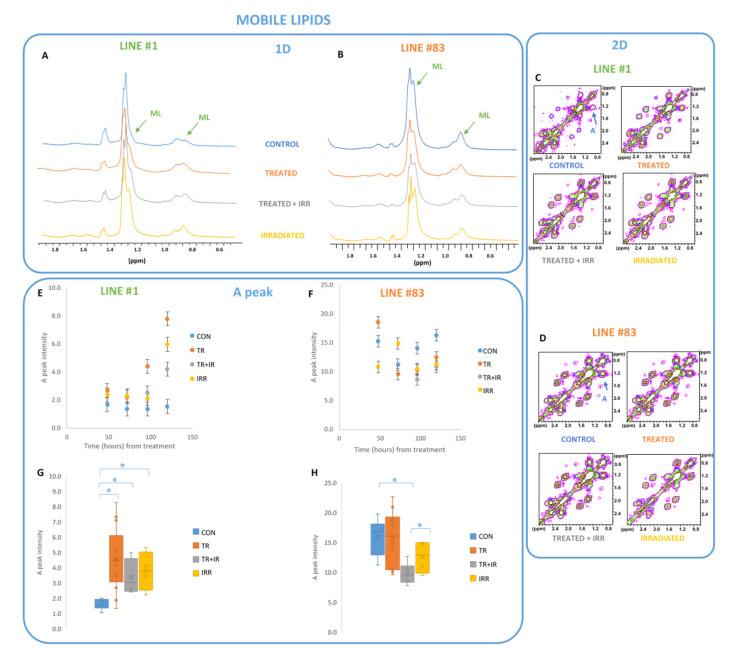
Mobile Lipid region of 1D (**A**,**B**) and 2D (**C**,**D**) spectra of cell line #1 (**A**,**C**) and line #83 (**B**,**D**) in the four different conditions (control—CON; ligand #190 treated—TR; photon beam irradiated—IR; and irradiated plus treated—IR+TR) 96 h after treatment. In the 2D contour plots (C and D), different colours indicate different intensity levels. The 2D COSY A peak (1.28–0.89 ppm) intensity, arising from mobile lipids CH_2n_ and terminal CH_3_ correlation, is shown as a function of time from ligand #190 treatment for line #1 (**E**) and line #83 (**F**). Box and whiskers plot comparing the A peak intensity values in the four different conditions for line #1 (**G**) and line #83 (**H**). Asterisks indicate statistically significant variation (Student’s *t*-test, *p* value < 0.05).

Student’s *t*-test performed on all the samples shows a statistically significant difference between controls and the three differently treated samples of line #1, while the three treatment conditions do not show significant variations between them (Figure 2G and Table 1). For line #83 the intensity variations of mobile lipids between controls and treated plus irradiated samples and between the latter and the irradiated samples are statistically significant (Figure 2H and Table 1). Other metabolites deserving attention are glutathione (GSH), glutamic acid (Glu), myo-inositol (Myo-I), N-acetyl-aspartate (NAA) and glutammine (Gln).

**Figure 3:**
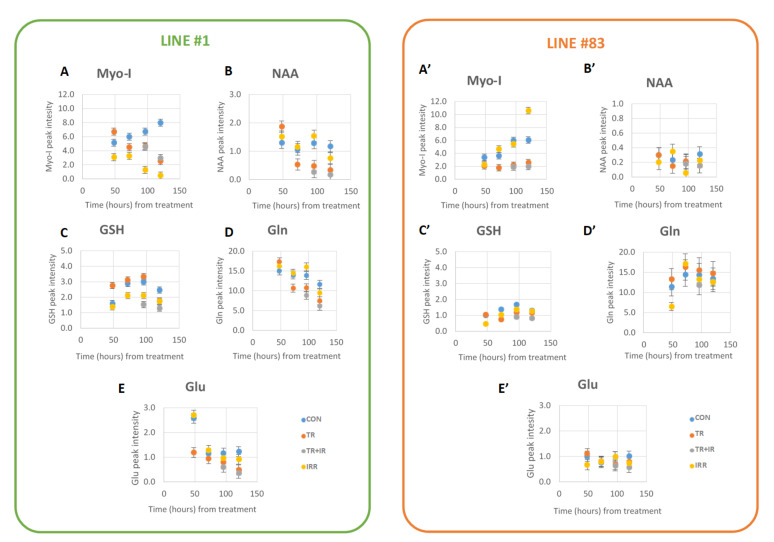
2D COSY peak intensity of metabolic signals from Myo-I at 3.61–3.27 ppm (**A**,**A’**), NAA at 2.67–2.48 ppm (**B**,**B’**), GSH at 2.56–2.15 ppm (**C**,**C’**), Gln at 2.44–2.13 ppm (**D**,**D’**), and Glu at 2.35–2.10 ppm, (**E**,**E’**) quantified in spectra from line #1 (**A**–**E**) and line #83 (**A’**–**E’**), respectively, as a function of time from ligand #190 treatment.

**Figure 4:**
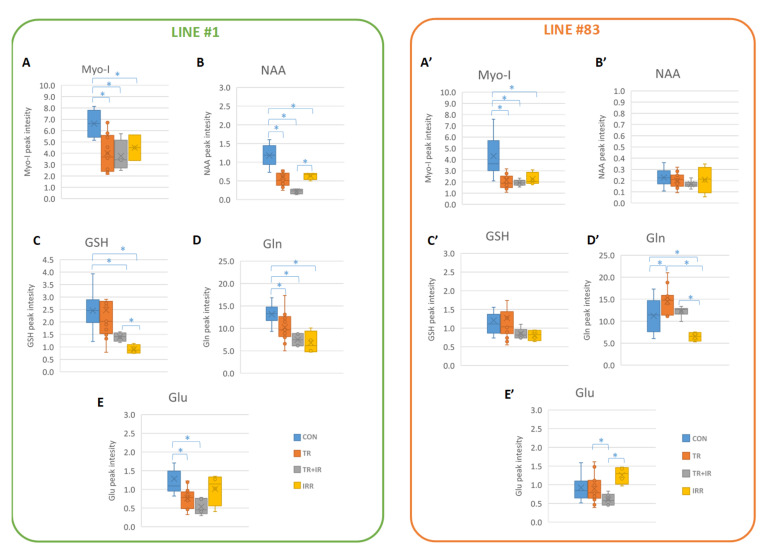
Box and whiskers plot comparing the peak intensity values for metabolites Myo-I (**A**,**A’**), NAA (**B**,**B’**), GSH (**C**,**C’**), Gln (**D**,**D’**), and Glu (**E**,**E’**) in the four different conditions for line #1 (**A**–**E**) and line #83 (**A’**–**E’**). Asterisks indicate statistically significant variations (Student’s *t* test, *p* value < 0.05).

Myo-I signals are present in a high amount in both cell lines’ control spectra; however, line #1 cells, characterized by a neural fingerprint, showed a higher Myo-I signal intensity (Figure 3A,A’ and Figure 4A,A’). Treated, irradiated and treated plus irradiated cell sample spectra showed a statistically significantly decreased concentration of Myo-I with respect to controls (Figure 4A,A’); as for ML signals, the three treatment conditions do not show significant variations between them (Figure 4A,A’ and Table 1).

The N-acetylaspartate (NAA) signal is clearly observable in line #1 cell spectra while it is slightly above the detection threshold in cell #83 spectra (Figure 3B,B’). Treatments induce a net decrease in NAA concentration in line #1 cell spectra (Figure 3B and Figure 4B), and a synergistic effect of ligand treatment plus irradiation seems to be present for this metabolic signal: a statistically significant difference between treated plus irradiated vs. irradiated samples was observed (Figure 4B and Table 1). No effects were induced by the different treatments in NAA signal intensity from line #83 spectra (Figure 3B’, Figure 4B’ and Table 1).

The signals of GSH, Gln, and Glu were influenced in line #1 and line #83 after treatments, though to a different extent (Figure 3 and Figure 4C,D,E,C’,D’,E’). In particular, in line #1, the intensity of all these signals decreases after the different treatments, whereas no statistically significant effects were observed for GSH in line #83, where this metabolite is present at a lower level (Figure 3C’ and Figure 4C’ and Table 1). Interestingly, a net effect of GSH decrease was observed when treating line #1 cells with radiation, and a synergistic effect of ligand treatment plus radiation is observable for this metabolite (Figure 4C). It is worth noting how the GSH, known as a powerful antioxidant, only comes into play after radiation treatment, while it does not vary significantly when dealing with the drug alone.

**Figure 5:**
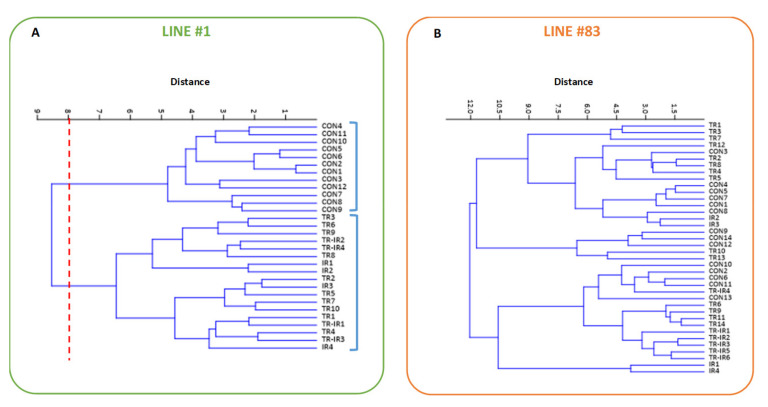
Clusters obtained from unsupervised hierarchical analysis for 10 metabolic parameters from 2D spectra (ML peaks A, B, and F; Myo-I, NAA, lactate, GSH-1; GSH-2; Gln and Glu, see Supplementary Figure S5B) of line #1 (**A**) and line #83 (**B**). CON—control samples; TR—ligand 190 treated samples; IR—irradiated samples; TR-IR—ligand 190 treated plus irradiated samples.

Finally, an unsupervised hierarchical analysis was performed with a metabolomics approach for both lines for the 10 metabolic parameters from 2D spectra (ML peaks A, B and F; Myo-I, NAA, lactate, two peaks from GSH; Gln and Glu, see Supplementary Figure S5B). The obtained clusters show that line #1 controls are grouped together into a different cluster with respect to treated samples, which, regardless of the treatment, are all grouped together (Figure 5A); instead, for line #83, all samples were classified in the same cluster (Figure 5B).

From the metabolic point of view, any synergistic effect between ligand #190 and radiation was observed. In addition, these results confirm that the mesenchymal line #83 is more resistant to radiation and treatment with radiosensitizer than line #1.

## 3. Discussion

A key challenge to GBM treatment is intratumoral heterogeneity at both cellular and microenvironmental levels [35,36]. Currently, a significant number of potential tumor-specific markers of different chemical natures have been identified, whose changes in qualitative and quantitative composition are associated with GBM progression. However, only a few of these identified markers have found application in clinical practice as prognostic or diagnostic markers due to the high heterogeneity of GBM [37]

At the single cell level, GBM is highly heterogeneous with a spectrum of stem cell and metabolic phenotypes [38,39], and contains both fast-cycling and slow-cycling cells that have distinct metabolisms and cancerous phenotypes [40]. Maintenance of heterogeneity may be driven by a GSC population within the tumor, such populations being highly plastic and responsive to their environment and holding self-renewal and tumor initiation capacities [41,42]. Different therapies have been proposed to target GSC heterogeneity.

Metabolomics may help in clarifying stem cell fate and reprogramming function [43,44]. In the present study, two GSC lines, namely, #83 and #1, characterized by different metabolic profiles [32] and clone heterogeneity [45] were analyzed as a response to photon beam treatment after previous treatment with G4-quadruplex ligand #190 in terms of synergistic effects on cell survival and metabolism. Results obtained on line #83 cell growth after single (ligand or irradiation) and combined (ligand + irradiation) treatment showed that:

(i) Line #83 was only slightly affected by irradiation, as previously observed in Palma et al. [12]. Ligand #190 induced a growth arrest; however, further cell irradiation did not induce any additional effect;
(ii) Line #1 cells showed a slowdown in growth after the combined treatments with radiation and ligand #190. Cell growth was mainly slowed down by the action of the ligand, while the combined treatment gave only a moderate synergistic effect;
(iii) MRS experiments on the two cell lines showed significant effects on several metabolites, mainly on lipid signals.

### 3.1. Lipid Metabolism in Gliomas

Lipid metabolism is abnormally regulated in gliomas compared to normal cells. GBM tumors accumulate more fatty acids than the surrounding normal brain [46,47]. These lipid stores, often used as an energy reservoir [48], can fuel GBM cell proliferation [49], and avoid oxidative damage and lipotoxicity [50]. Recently, lipid metabolism has emerged as a potential therapeutic target in the treatment of gliomas, including GBM [46,51,52]. These lipid stores, organized as lipid droplets, are cytosolic organelles that, among other functions, serve as a storage medium for the fatty acids, protecting the cell from oxidative damage and providing an energy source to maintain proliferation in stressful conditions, such as hypoxia and nutrient deprivation [53]. Accumulations of lipid droplets have been observed in a variety of cancers, including hepatic, lung, and breast cancer, as well as gliomas, and they are an important regulator of critical events, including angiogenesis, inflammatory responses, apoptosis and cell death, cancer metastasis, and hypoxia-mediated alterations of lipid metabolism [54]. Glioma stem cells rely mainly on oxidative phosphorylation [55]. Lipid metabolism has been regarded as one of the key factors for the correct function of pathways involved in GSC fate and characteristics, e.g., chemotherapy evasion [56,57]. Enhanced lipid metabolism is essential for the survival, growth, and oncogenicity of GSCs [58,59]. GSCs seem to have increased levels of lipids and fatty acid oxidation (FAO)-related genes and these can maintain GSC self-renewal by modulating lipid and membrane synthesis, quenching ROS through NADPH production, and promoting chemo-resistance [23,59].

From the MR spectra of the two cell lines, we observed that the mobile lipid content of control samples present in cytoplasmic droplets is quite different from the cell lines and is also differently influenced by treatments, i.e., radiation [12]. In fact, statistically significant effects on A lipid peak from 2D spectra of irradiated cells are observed for line #1 with respect to controls; this increase was higher after ligand #190 treatment. Combined radiation and ligand #190 did not increase the effect on the lipid signal. In line #83 cells, a statistically significant decrease of the lipid signal with respect to control was observed only after the combination of radiation plus drug treatments. In line #1, single and combined treatments produced similar effects on lipids. On the contrary, in line #83 cells, known to be less sensitive either to radiation or G-quadruplex compound treatment [12,23], the combined treatment induced a net decrease of these signals. This suggests that photon irradiation and ligand #190 treatment are characterized by different mechanisms of action on the two cell lines. Metabolism impairment may be invoked to justify the accumulation of lipids in the cytoplasm detected as the ML increase in line #1 [60,61], whereas, in line #83, MLs, segregated and stored mainly in cytoplasmic lipid droplets, seem to be protected from oxidation and from the formation of unstable lipid peroxides.

### 3.2. Other Metabolic Changes in Glioma Cells

Metabolic alterations have long been regarded as a hallmark of cancer cells; however, contradictory results have been reported for GSCs, suggesting metabolic plasticity, especially concerning mitochondria and energetic metabolism [62]. Recent studies showed different metabolic phenotypes of GSCs, identifying two clusters of GSCs in the murine GBM model, Clone A and Clone B. Cells of Clone A are glycolysis dependent, while the metabolic phenotype of Clone B can switch between mitochondrial respiration and glycolysis [63]. Analogously, in a previous study, we identified similar behavior in classifying a panel of GSCs in two different clusters [44].

Glutamine (Gln)-high GSCs metabolize more glutamine to sustain mitochondrial respiration, and the reduction of glutamine can weaken their ability to enhance cell proliferation and self-renewal. Our study on genetic and metabolic profiles distinguished two clusters of GSCs, GSf-like and GSr-like [33,34]. Cells of the former group, which line #1 belongs to, show metabolic features with low mobile lipids and high glutamine levels, while cells in the latter group, including line #83, show the opposite. Activated glutamine metabolism is also associated with GSC resistance to radiotherapy. Therefore, therapy-resistant GSCs consume less glucose but activate glutamine and lipid metabolism.

It is known that GBM tissue shows a higher glutamine level than the surrounding normal brain and that the ability to metabolize glutamine is critical for GBM proliferation and survival [12]. In cell line #1, Gln levels decrease over time in culture, both in control and in treated cells. This cell line is affected by radiation or by ligand #190 treatment with a net decrease of the Gln signal. Line #1 cells showed a similar response to radiation and ligand #190, but no synergism in the combined treatment. On the contrary, line #83 cells maintain high levels of Gln without any decrease over time, suggesting different pathways for Gln consumption [12]. In cell line #83, Gln levels decreased after irradiation, while they showed an opposite behavior after single ligand and combined treatments. This peculiar behavior may be likely ascribed to the higher heterogeneity in this cell line [64].

Glu is often synthesized from glutamine by the enzyme glutaminase or from oxidative deamination. Its synthesis and degradation are part of the glutamate/glutamine cycle. Due to its role as a neurotransmitter, Glu is constantly released from neurons and its uptake by astrocytes at the neuronal synapse is essential for maintaining normal brain function [65,66]. After its release, Glu is taken up by astrocytes, where it is converted to Gln and released again [64]. In glioblastoma, glutaminolysis serves as an energy fuel and seems to be a prerequisite for tumor cell growth. An increased glutamine level in gliomas has also been demonstrated by using high resolution proton MR spectroscopy in vitro [67] and has even led to the description of gliomas as “glutamine traps”. GBM tumors have been observed to take up more glutamine than the surrounding normal brain tissue in vivo [68]. Both cell lines #1 and #83 showed a low level of glutamate (Figure 3 and Figure 4) and Glu metabolism seems slightly affected in both lines. Interestingly, in line #1 spectra Glu levels decreases after ligand plus radiation treatments. On the contrary, in line #83 spectra no statistically significant effects were observed. The decrease of Glu in line #1 spectra parallels the decrease of Gln signals, assuming a flux of Glu in favor of Gln reserves for energetic metabolism. In line #83 cells, a different mechanism seems to work. Further efforts to explore the mechanisms of how abnormal metabolic patterns affect cell resistance to therapy are needed.

### 3.3. Glutatione (GSH) Metabolism in Gliomas

Molecular changes in the GSH antioxidant system and remolding in GSH homeostasis have been implicated in tumor initiation, progression, and treatment response because of both protective and pathogenic roles of GSH. Although in healthy cells it is crucial for the removal and detoxification of carcinogens, elevated GSH levels in tumor cells are associated with tumor progression and increased resistance to chemotherapeutic drugs. Recently, several novel therapies have been developed to target the GSH antioxidant system in tumors [69].

GSH deficiency or a change in the GSH/GSSG ratio increases the vulnerability of cells to oxidative stress, inflammation, and tumor progression. However, elevated GSH levels increase antioxidant capacity and resistance to oxidative stress, as is evident in many tumors [69]. It has been shown that exogenous addition of GSH inhibits the inflammatory response through regulation of ROS, while endogenous GSH has been recently indicated to play a role in fine-tuning the innate immune response to infection, thereby regulating inflammation [70]. GSH, therefore, has a dual role in the inflammatory response, as an antioxidant ROS scavenger in the oxidative stress as well as a signaling molecule.

As for GSH, signal intensities in spectra from line #83 are not affected by treatments, thus suggesting that GSH is not a key metabolite in protecting cells from treatment; other mechanisms have to be invoked probably due to the high heterogeneity of line #83 and cell lines belonging to the mesenchymal cluster. In addition, GSH levels in line #83 are low and do not seem to be relevant for cell protection. [12] On the contrary, in line #1, GSH levels noticeably decrease after both radiation and ligand #190 treatment plus radiation with a synergistic effect, while no significant effects are observable with ligand #190 alone. This could be attributed to the antioxidant properties of GSH, which decreases to protect cells against the free radicals produced by irradiation [12].

### 3.4. Myo-I Metabolism in Gliomas

Myo-I is one of the most abundant metabolites in the human brain, and is located mainly in glial cells and functions as an osmolyte. Myo-I concentration is high in many brain disorders, including Alzheimer’s disease and in a variety of pathological conditions that involve astrocytic proliferation [67,71]. The myo-inositol MRS signal was also affected by treatments in a significant way and it is present in high amounts in both cell lines, however, line #1 cells, characterized by a neural fingerprint [11], showed a high Myo-I signal intensity. Ligand-treated, radiated and ligand-treated plus radiated cells showed a decreased concentration of Myo-I. In our experiments, higher levels of Myo-I were observed in line #1 cells with respect to line #83. In both cell lines, Myo-I was affected by either radiation alone, or ligand alone, or combined radiation and ligand. This result supports the hypothesis that the effect can be attributed to a non-specific response.

### 3.5. Metabolism of NAA in Gliomas

As far as NAA is concerned, the literature reports that NAA levels decreased in GBM compared to normal brains and that NAA is a marker of the neuronal phenotype. Different levels of NAA were observed in GSCs associated with different metabolic and genomic signatures [33,44]. In malignant and benign brain tumors, NAA is markedly decreased or absent, suggesting axonal loss. Apparently, the absence of NAA biosynthetic enzyme (aspartate N-acetyltransferase) in brain tumors is the main cause of NAA signal loss. We observed that the NAA signal in line #83 cells is barely detected; on the contrary, NAA seems noticeably affected in line #1 cells with a likely synergistic effect after the combined treatment.

An unsupervised statistical analysis of 10 metabolites (A, NAA, GSH, Glu + GSH, Myo-I, F, Gln, Glu, and Lac), made with a metabolomic approach, showed different groupings for the two cell lines. In line #1, controls are grouped in a different cluster compared to their treated counterparts (which, regardless of the treatment, are all grouped together), while for line #83 cells, no cluster separation was observed.

In conclusion, in both cell lines a possible synergistic interaction of combined treatments seems to be excluded. From the metabolic point of view only for a few metabolites was a synergistic effect between ligand treatment and radiation observed, but the complete statistical analysis does not confirm this synergy. These results confirm that the mesenchymal phenotype is more resistant to radiation and treatment with ligand #190 than the proneural phenotype. Specifically tailored therapies should be developed which take into account the high heterogeneity of GSCs as the use of combined treatments, such as those proposed in this paper, seem to fail. On the other hand, single treatment with G-quadruplex stabilizing ligand seems to induce alterations at the metabolic level in the two GSC lines, mainly in the proneural phenotype.

## 4. Materials and Methods

### 4.1. Ligand #190 Synthesis

All solvents and reagents were purchased from TCI and Sigma-Aldrich and used without further purification. HPLC analyses were performed using an Agilent system SERIES 1260 (Agilent Technologies, Santa Clara, CA, USA) with an XBridge^®^ BEH C18 column (2.5 μm, 4.6 × 50 mm). The following method was used: flow 1.4 mL/min, isocratic gradient over 2 min 95% of H_2_O + 0.1% TFA (5% CH_3_CN), gradually to 40% aqueous solvent over 6 min, then isocratic flow for 4 min (λ = 256 nm). UPLC-MS data were recorded using a surveyor UPLC system (Thermo Finnigan, San Jose, CA, USA) equipped with a BEH Acquity UPLC column (1.7 μm) 2.1 × 50 mm, and an LCQ ADV MAX ion-trap mass spectrometer, with an ESI ion source. Chromatographic purification was conducted using ISOLERA-One, a Biotae Flash Purification system. Recordings of 1H-NMR spectra were made with a Bruker Avance 300 MHz (Bruker, Billerica, MA, USA) calibrated to the internal standard TMS or the residual solvent peak. All the products were characterized by ESI-MS and 1H-NMR. The synthetic procedure needed to obtain RHPS4 derivative is reported in detail in the Supplementary Materials and Figures S1–S3.

### 4.2. GSC Isolation and Cell Culture

GSCs were obtained from adult GBM patients (WHO grade IV) who had undergone complete or partial surgical resection at the Institute of Neurosurgery, Catholic University School of Medicine, Rome, Italy. Informed consent was obtained from the patients before surgery. The tumor tissues were mechanically dissociated and single-cell suspension cultured in a serum-free medium supplemented with epidermal growth factor and basic fibroblast growth factor, as previously described [72]. The in vivo tumorigenic potential of GSCs was assessed by intracranial cell injection in immunocompromised mice, where GSCs were capable of recapitulating the patient tumor in terms of antigen expression and histological tissue organization. GSC lines were validated by short tandem repeat (STR) DNA fingerprinting, as previously described [73]. Clones from GSCs were obtained by seeding single cells onto a 96-well plate. After 4 weeks, single clones were mechanically dissociated and reseeded to expand the culture.

### 4.3. Ligand #190 and Irradiation Treatment

For ligand #190 dose-response experiments, GSCs were mechanically dissociated and plated in 96-well plates at a density of 2 × 10^4^ cells/mL. After 16 h, #190 was added to the cells. ATP levels were measured at different time points as a surrogate of cell viability using CellTiter-Glo™ (Promega Inc., Madison, WI, USA) according to the manufacturer’s instructions. The mean of the raw luminescence values from triplicate wells treated with vehicle alone (mL_C_) was used as a reference to interpolate percentage viability from wells treated with drugs (V_D_), using the following formula: V_D_ = (L_D_/mL_C_) × 100 [74].

Maximal inhibitory concentration (IC50) and 25% inhibitory concentration (IC25) values were determined by a non-linear regression curve through the plotted experimental data and represent compound concentrations able to reduce cell proliferation by 50% and 25%, respectively.

For MRS and cell viability experiments, GSCs were seeded in T175 cm^2^ flasks at a density of 10 × 10^4^ cells/mL in 50 mL total volume. Cells were the treated with 0.7 µM (line #1) and 0.75 µM (line #83) ligand #190 concentration (IC 25 for each line) for 48, 72, 96, and 120 h.

For combined treatments (see Supplementary Figure S4), cells were seeded as described previously, treated with ligand #190 (IC 25) and, after 72 h, irradiated in culture flasks at a single acute dose of 20 Gy with a cesium-137 (^137^Cs) gamma ray source (Gammacell 40 Exactor, NORDION, Ottawa, ON, Canada) operating at a dose rate of 0.8 Gy/min (Istituto Superiore Sanità facility). A single radiation dose of 20 Gy was used, comparable to total doses delivered during radiation therapy in a fractionated regimen and to a single high dose used in special therapeutic modalities, such as intraoperative radiotherapy and stereotactic radiosurgery [75].

### 4.4. H MRS Cell Sample Preparation

Cells were washed in phospate buffered saline (PBS) (Corning, NY, USA) without calcium and magnesium at pH 7.4 and centrifuged at 162 rcf for 3 min. The pellet was resuspended in PBS with 20% D_2_O and 2mM Sodium 3-(trimethylsilyl)propionate-2,2,3,3-d4 (TMSP) as a frequency standard. A 20 μL aliquot of the suspension was transferred into a 1 mm MR tube and centrifuged to obtain a packed cell volume, as previously described [32]. All MRS reagents were purchased from Cambridge Isotope Laboratories, Inc., Tewksbury, MA, USA.

### 4.5. H MRS Measurements

^1^H MRS experiments were run on a digital Bruker Avance spectrometer (Bruker Scientific LLC, Billerica, MA, USA) at 400.14 MHz equipped with a 1mm microprobe. Both one-dimensional (1D) and two-dimensional correlation spectroscopy (2D COSY) experiments were performed, at T = 298 K.

1D ^1^H MRS spectra of GSCs and culture media were acquired with a 90° RF pulse, the number of scans (ns) was equal to 1000 (sufficient to obtain a good signal-to-noise ratio) for cell spectra. 2D COSY spectra were acquired with a 90°-t1–90°-t2 pulse sequence and ns = 32 for cell or ns = 128 for culture media samples. Spectra were acquired as a matrix of 512 × 128 data points in time domain.

MRS parameters were obtained in at least three independent experiments and data expressed as mean ± standard deviation (SD) values. WINNMR software (Bruker Scientific LLC, Billerica, MA, USA) was used to perform 1D signal deconvolution and 2D cross peak integration, as reported in [32]. A macromolecule signal at 0.89 ppm intensity was used as internal reference for 1D measurement, while 2D signal integrals were normalized to the intensity of the lysine (Lys) cross-peak at 1.70–3.00 ppm. This peak was considered representative of the cellular mass, as it was found to be constant in a number of cell models and tissue samples [32].

### 4.6. Statistical Analysis

Unsupervised agglomerative hierarchical clustering, principal component analysis and Student’s *t*-test were performed utilizing Past 4.03 Software, version 2020 [76].

## Figures and Tables

**Table 1:** Student’s *t*-test *p* values for the indicated metabolites between different condition samples.

***p*-Value**						
**Line #1**	**A**	**Myo-I**	**GSH**	**NAA**	**Gln**	**Glu**
CON-TR	0.0002	0.0002	0.9608	0.0007	0.0134	0.0032
CON-TR+IR	0.0039	0.0010	0.0122	0.0001	0.0002	0.0071
CON-IR	0.0010	0.0130	0.0008	0.0030	0.0002	0.3438
TR-TR+IR	0.2873	0.7910	0.3530	0.0808	0.1213	0.0706
TR-IR	0.4845	0.6515	0.0540	0.9201	0.0677	0.1829
IR-TR+IR	0.6508	0.4907	0.0076	0.0004	0.6543	0.0599
*p*-Value						
**Line #83**	**A**	**Myo-I**	**GSH**	**NAA**	**Gln**	**Glu**
CON-TR	0.8213	0.0005	0.7732	0.3474	0.0184	0.8406
CON-TR+IR	0.0001	0.0050	0.0815	0.0797	0.5177	0.0337
CON-IR	0.0506	0.0415	0.1096	0.6933	0.0258	0.0689
TR-TR+IR	0.0056	0.5769	0.1536	0.2222	0.1070	0.0743
TR-IR	0.2069	0.8341	0.1990	0.8991	0.0001	0.0766
IR-TR+IR	0.0795	0.2535	0.6462	0.4463	0.0001	0.0006

## Data Availability

All the data and materials used and/or analyzed during this study are available from the corresponding authors on reasonable request.

## Supplementary Materials

The following are available online at https://www.mdpi.com/article/10.3390/ijms222312709/s1.
