# Supplementary material for: Effects of the Combined Treatment with a G-Quadruplex-Stabilizing Ligand and Photon Beams on Glioblastoma Stem-like Cells: A Magnetic Resonance Study
Source: Int J Mol Sci. 2021 Nov 24;22(23):12709. doi: 10.3390/ijms222312709 (PMC8657890; doi:10.3390/ijms222312709)
Supplement: Supplementary file 1 [file ijms-22-12709-s001.zip › Supplementary Figure S4.pdf]

Supplementary Figure 4.

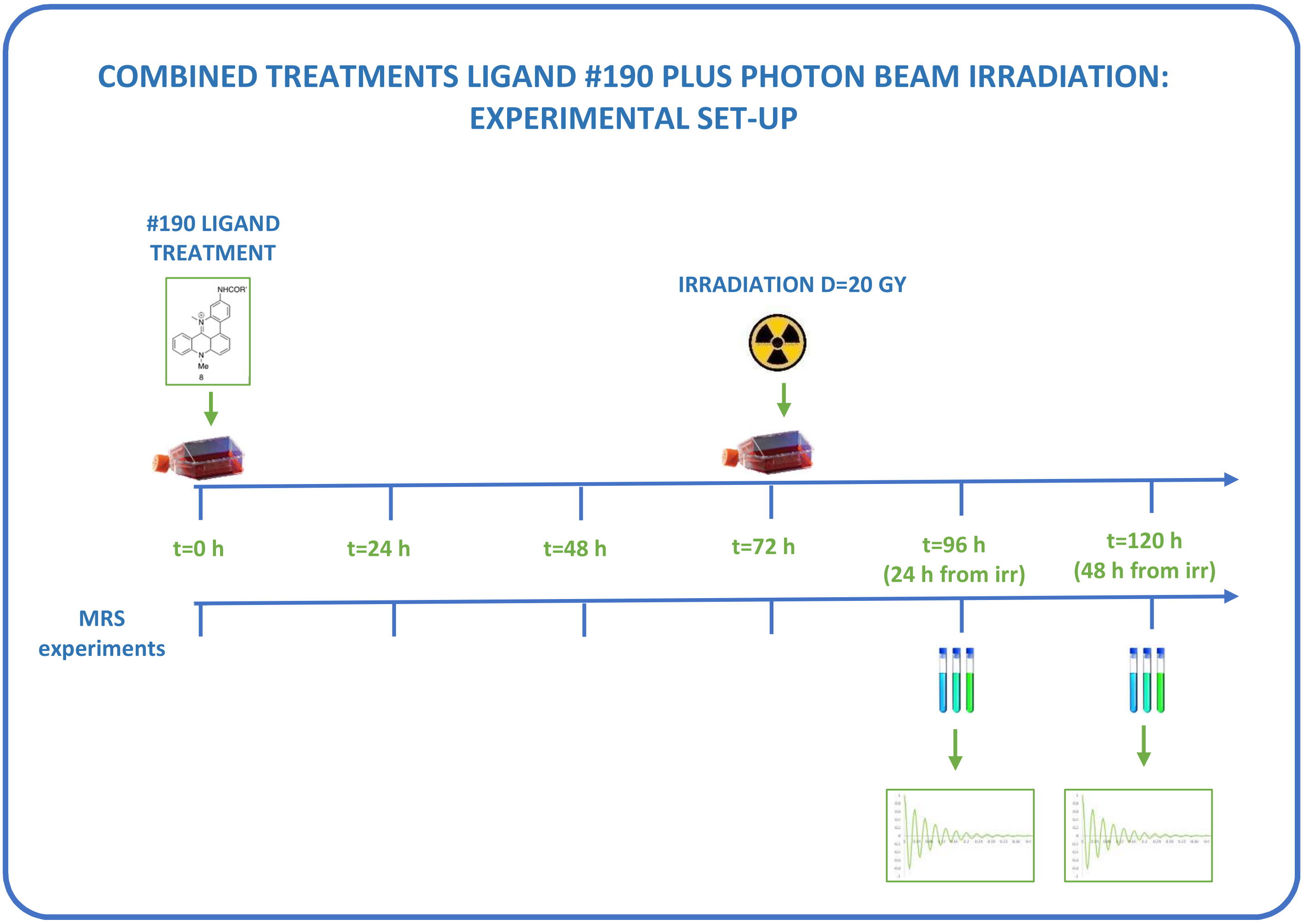

Figure S4. Scheme representing the time line of the experimental set-up of combined treatment MR experiments.
