# Supplementary material for: Effects of the Combined Treatment with a G-Quadruplex-Stabilizing Ligand and Photon Beams on Glioblastoma Stem-like Cells: A Magnetic Resonance Study
Source: Int J Mol Sci. 2021 Nov 24;22(23):12709. doi: 10.3390/ijms222312709 (PMC8657890; doi:10.3390/ijms222312709)
Supplement: Supplementary file 1 [file ijms-22-12709-s001.zip › Supplementary Figure S5.pdf]

Supplementary Figure 5.

A: 1D ^{1}H MR spectra of line #1 and line #83 control cells at day 4 after seeding. In the lower panels, a Lorentzian-Gaussian function was applied for resolution enhancement with LB=-10 and GB=15. Assignments were performed according to Supplementary References [1,2].

B: Exemplificative 2D COSY spectra of a line #83 control sample at day 4 after seeding. The ten cross peaks used for the analysis are labelled. Assignments (see Table in the Figure S5 B) were performed according to Supplementary References [1,2]. The lysine (Lys) cross-peak at 1.70–3.00 ppm was used as intensity reference. Mobile lipid correlation A, B and F are shown in the inset.

Supplementary References

1 Guidoni L, Ricci-Vitiani L, Rosi A, Palma A, Grande S, Luciani AM, Pelacchi F, Di Martino S, Colosimo C, Biffoni M, De Maria R, Pallini R, Viti. 1H NMR detects different metabolic profiles in glioblastoma stem-like cells. *NMR Biomed*. **2014**; 27: 129–145

2 Govindaraju V, Young K, Maudsley AA. Proton NMR chemical shifts and coupling constants for brain metabolites. *NMR Biomed.* **2000** May;13(3):129-53. doi: 10.1002/1099-1492(200005)13:3<129::aid-nbm619>3.0.co;2-v.

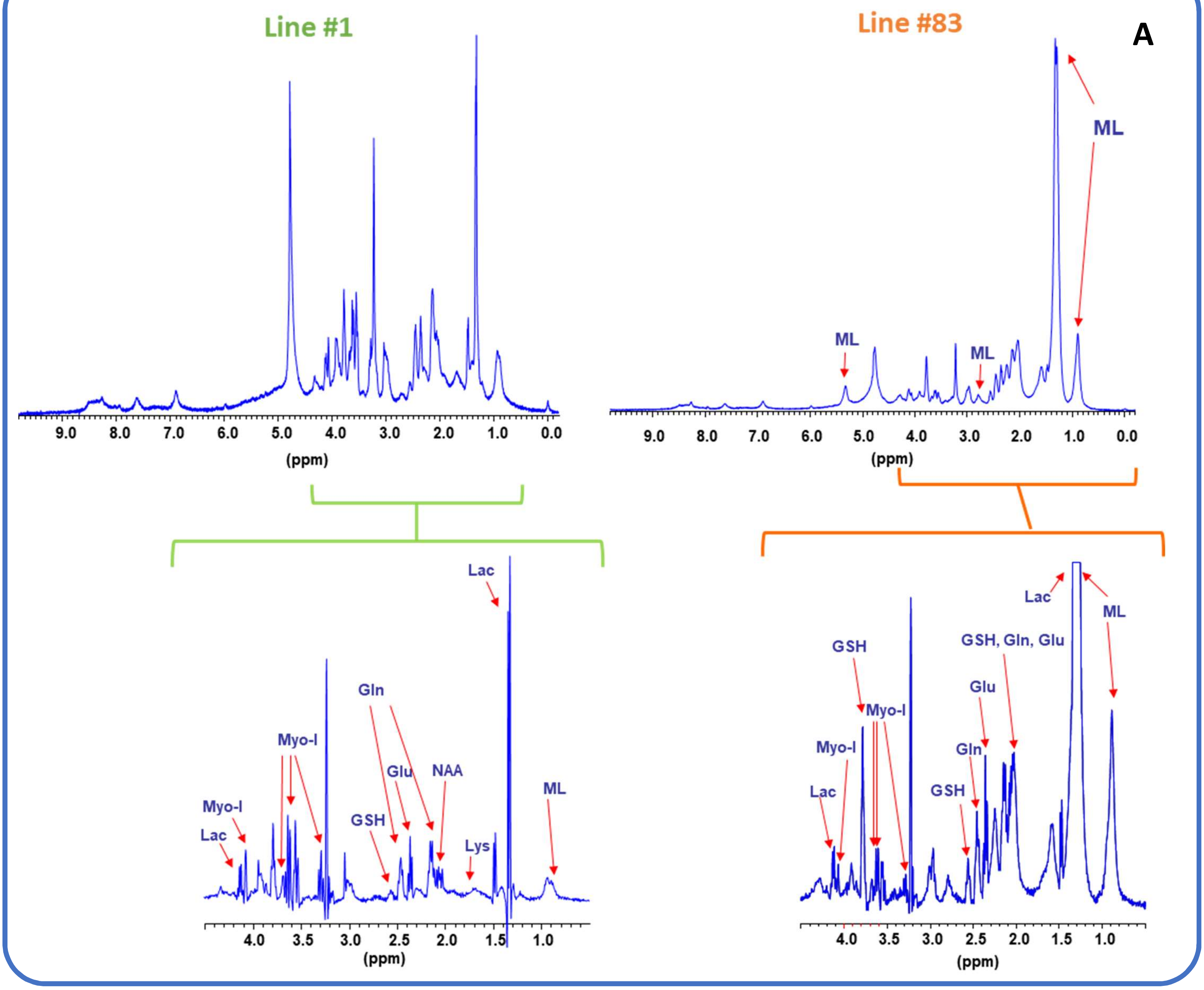

B

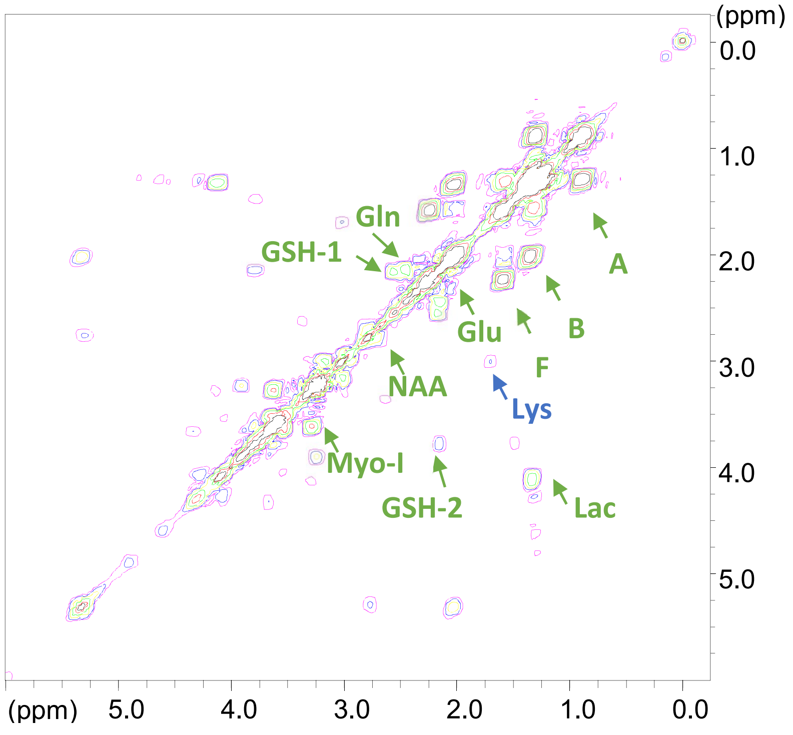

Cross peak	Chemical shift (ppm)
A	1.28-0.89
B	2.02-1.28
F	2.35-1.65
Lac	4.11-1.33
Glu	2.35-2.10
Gln	2.44-2.13
GSH-1	2.56-2.15
GSH-2	3.77-2.15
NAA	2.67-2.48
Myo-I	3.61-3.27
Lys	3.00-1.70

Mobile Lipid chain

F B A

$HOOC\text{-}CH_2\text{-}CH_2\text{-}(CH_2)_4\text{-}CH_2\text{-}CH{=}CH\text{-}CH_2\text{-}CH{=}CH\text{-}CH_2\text{-}(CH_2)_2\text{-}CH_2\text{-}CH_3$

2.35 1.65 1.3 2.05 5.3 5.3 2.80 5.3 5.3 2.05 1.3 1.28 0.89
