# Supplementary material for: Effects of the Combined Treatment with a G-Quadruplex-Stabilizing Ligand and Photon Beams on Glioblastoma Stem-like Cells: A Magnetic Resonance Study
Source: Int J Mol Sci. 2021 Nov 24;22(23):12709. doi: 10.3390/ijms222312709 (PMC8657890; doi:10.3390/ijms222312709)
Supplement: Supplementary file 1 [file ijms-22-12709-s001.zip › Supplementary Materials_and Figures_S1-S3.pdf]

Supplementary Materials and Figures S1-S3

Synthesis of RHPS derivative ***8,*** **Compound #190**

The first 6 products were synthesized slightly modifying already known procedures (1, 2 3) to increase the yields of the reactions. The last two protocols are based on known strategy but never reported on these substrates in literature (3). For the complete schematic representation of the synthetic protocols utilized, please refer to Figure 1A.

Synthesis of 9-(2-Amino-5-nitroanilino)- acridine hydrochloride **2:**

The 2-amino-4-nitroaniline (1 eq, 71.7 mg) was dissolved in methanol (3 mL), and treated with 9-chloroacridine (1 eq, 100 mg) over 30 min. This mixture was refluxed for 90 min, checked in TLC (6:4 Esane: EtOAc), cooled and diethyl ether (1 mL) was added to induce the precipitation of the desired product. The anilinoacridine hydrochloride **2** precipitated was filtered on Buchner, washed with diethyl ether, and collected (88.7 mg, 57% yield). UPLC-MS, positive mode: 331.12 [**2**-H]$^+$ *m/z*. ^{1}H NMR (300 MHz, DMSO) δ_H 11.06 (br s, 1H, NH), 8.28 (d, 2H), 8.14 (m, 2H), 8.09 (d, 2H), 7.99 (m, 2H), 7.46 (t, 2H), 7.14 (br s, 2H, NH2), 6.98 (d, 1H).

Synthesis of 9-(6-Nitrobenzotriazol-1-yl)acridine **3:**

9-(2-Amino-5-nitroanilino)- acridine hydrochloride **2** (1 eq, 70 mg) was suspended in 2 M hydrochloric acid (2 mL) at 0–5°C and it was treated for 30 min with a previously prepared solution of sodium nitrite (0.0292 g, 2 eq.) in water (208.8 µL). The suspension was stirred at 0°C for 5 h, increasing the yield of the reaction checking the progression in TLC 8:2 Esane: EtOAc. In the end, the mixture was basified in a solution of aqueous ammonia–ice, and product **3** was filtered and collected as red solid (60%). UPLC-MS, positive mode: 342.09 [**3**-H]$^+$ *m/z*. ^{1}H NMR (300 MHz, DMSO) δ_H 8.91 (s, 1H), 8.35 (m, 3H), 8.25 (d, 1H), 8.01-7.85 (m, 6H).

Synthesis of 2-Nitro-8H-quino[4,3,2-kl]acridine **4:**

9-(6-Nitrobenzotriazol-1- yl)acridine **3** (43.3 mg, 1eq) was boiled for 4 h in a properly Triglyme amount needed to dissolve it. The mixture was allowed to cool to room temperature and water was added to obtain the 2-Nitro-8H-quino- [4,3,2-kl]acridine **4** as a red pure precipitate in 82% yield. UPLC-MS, positive mode: 316.10 [**4**-H]$^+$ *m/z*. ^{1}H NMR (300 MHz, DMSO) δ_H 11.82 (br s, 1H, NH), 8.24 (m, 2H), 8.14 (d, 1H), 7.90 (dd, 1H), 7.75 (m, 2H), 7.54 (t, 1H), 7.20 (m, 3H).

Synthesis of 8-Methyl-2-nitro-8H-quino[4,3,2-kl]acridine **5**:

A suspension of sodium hydride (5.9 mg, 2.6 eq) in DMF (1 mL) was treated with 2-Nitro-8H-quino[4,3,2-kl]acridine **4** (30 mg, 1 eq) previously dissolved in DMF (2 mL). The mixture was stirred for 1 hour at 25°C, then dimethylsulfate (17.9 mg, 1.5 eq, d 1.44 g/mL, 13.5 µL) was added dropwise, maintaining the mixture under stirring for further 4 h. The reaction mixture was poured into water (5 mL) and the precipitate was collected by filtration and washed with water (10 mL) to give compound **5** as a dark red solid. Yield 80%. UPLC-MS, positive mode: 330.12 [**5**-H]$^+$ *m/z*. ^{1}H NMR (300 MHz, DMSO) δ_H 8.67 (1H, d), 8.61 (1H, d), 8.42 (1H, d), 8.11–8.05 (2H, m), 7.86 (1H, t), 7.68–7.55 (2H, m), 7.45 (1H, d), 7.24 (1H, t), 3.67 (3H, s, CH_3).

Synthesis of 2-amino-8-methyl-8H-quino[4,3,2-kl]-acridine **6:**

A mixture of 8-methyl-2-nitro-8H-quino[4,3,2-kl]acridine **5** (200 mg, 1 eq) and $SnCl_2$
^2H_2O (0.6094 g, 4.42 eq) in 10 M HCl (5 mL) was stirred at room temperature for 5 days. After, NaOH 10 M was added to basify the solution until pH 12, and the resulting precipitate was filtered and washed with water yielding 2-amino-8-methyl-8H-quino[4,3,2-kl]-acridine **6** as a brown–red powder. Yield 79%. UPLC-MS, positive mode: 300.14 [**6**-H]+ *m/z*. ^{1}H NMR (300 MHz, MeOH) δ_H 8.72 (1H, dd), 8.19 (1H, d), 7.87 (1H, d), 7.71 (1H, t), 7.51–7.63 (2H, m), 7.22 (1H, t), 7.12 (1H, d), 6.99 (1H, d), 6.88 (1H, dd), 5.61 (2H, s, NH2), 3.66 (3H, s, CH_3).

Synthesis of N-(8-methyl-4b1,7a-dihydro-8H-quinolino[4,3,2-kl]acridin-2-yl) cyclopropanecarboxamide **7:**

2-amino-8-methyl-8H-quino[4,3,2-kl]-acridine **6** (1.0 eq, 490 mg) was dissolved in DCM (8.25 mL) at 0°C and cyclopropanecarbonyl chloride (1.0 eq, 150 µl), triethylamine (1.0 eq, 230 µl), and catalytic DMAP (0.03 g) were added. The mixture was stirred overnight, then evaporated to dryness. To induce compound **7** precipitation, 20 mL of water were added, and the red solid was collected by filtration. Yield 68%. UPLC-MS, positive mode: 368.15 [**7**-H]$^+$ *m/z*. ^{1}H NMR (300 MHz, $CDCl_3$) δ_H 8.50 (1H, dd), 8.29 (1H, s), 7.77 (5H, m), 7.39 (3H, dd), 3.83 (3H, s, CH_3), 1.86 (1H, t), 1.29 (2H, m), 1.00 (2H, m).

Synthesis of 2-(cyclopropanecarboxamido)-8,13-dimethyl-4b1,7a-dihydro-8H-quinolino[4,3,2-kl] acridin-13-ium **8:**

To obtain the final product, the 8-methylacridine **7** (0.1 mmol) was heated with excess methyl iodide (2 mL) for 4 days at 150°C in a pressure tube. The product was collected by filtration, washed with Et_2O, and purified by column chromatography (5–10% MeOH–$CHCl_3$). Yield 35%. UPLC-MS, positive mode: 383.19 [**8**-H]$^+$ *m/z*. ^{1}H NMR (300 MHz, MeOH) δ_H 8.72 (1H, dd, J 1.3 and 7.9), 8.51-8.35 (3H, m), 8.17 (dd, 4H) 7.85 (1H, t), 7.65 (2H, d), 4.35 (3H, s, CH_3), 4.15 (3H, t, CH_3), 1.92 (1H, t), 1.29 (2H, m), 1.00 (2H, m).

HPCL purity analysis, ^{1}H MR spectrum and ESI-MS mass spectrum of the purified Compound #190 are reported in Figures S1, S2 and S3 respectively.

HPLC Purity Analysis

Compound #190 (Rt = 7.09 min; 88.4%)

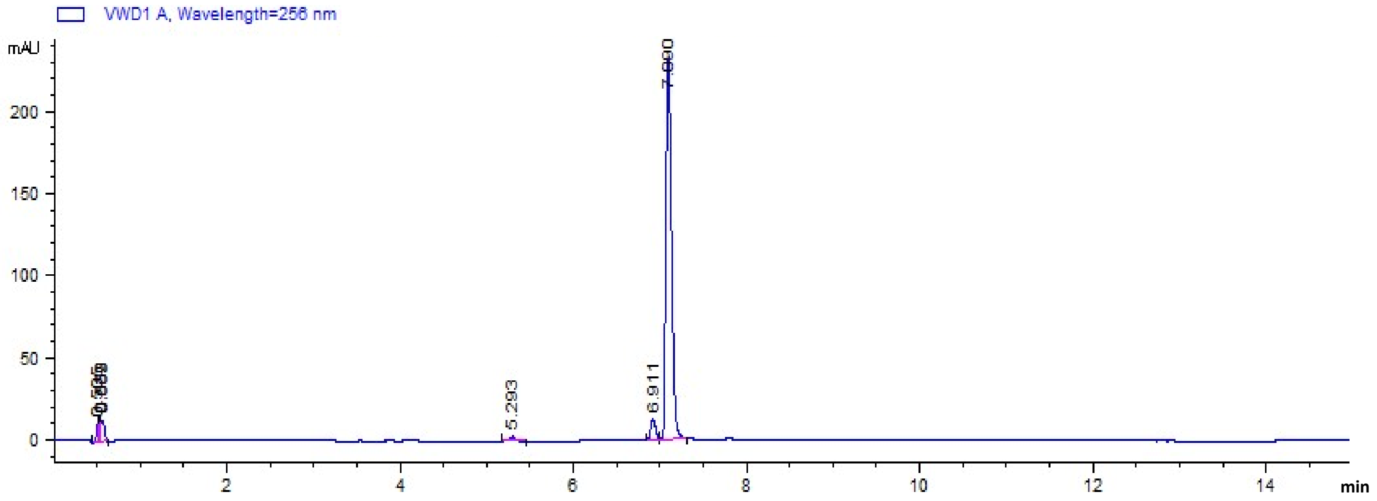

Figure S1. Chromatographic profile of **Compound #190** using following method: flow 1.4 mL/min, isocratic gradient over 2 min 95% of H2O + 0.1% TFA (5% CH3CN), gradually to 40% aqueous solvent over 6 min, then isocratic flow for 4 min (λ = 256 nm).

[1]H-MR Characterizations

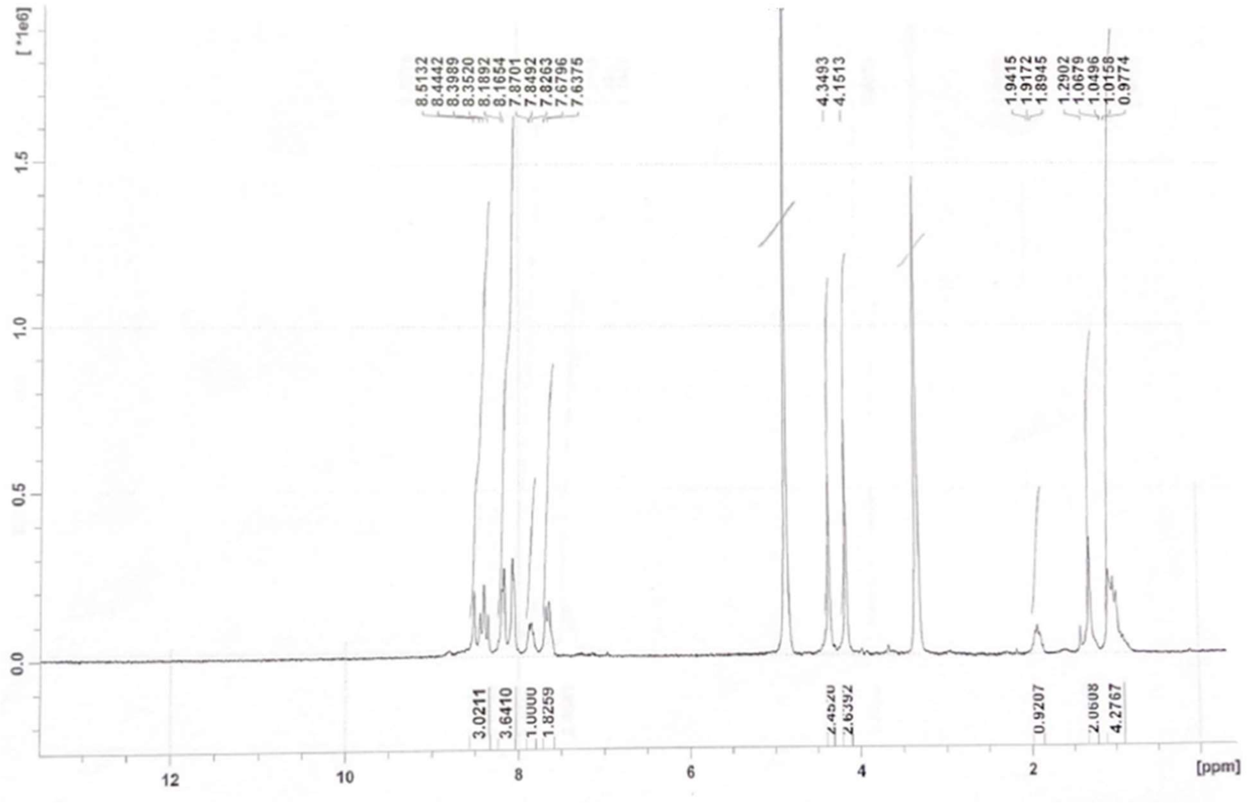

Figure S2. ^{1}H MR (300 MHz, MeOH) of **Compound #190**.

ESI-MS Data

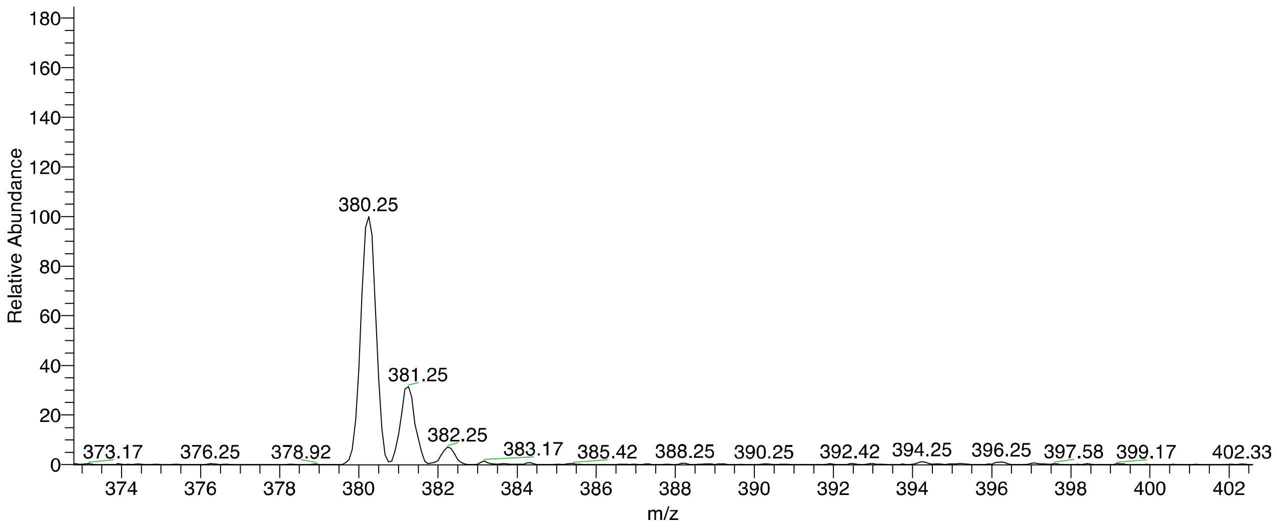

Figure S3. ESI-MS mass spectrum of the purified **Compound #190**.

Supplementary references

1. Stanslas Johnson, Hagan Damien J., Ellis Michael J., Turner Claire, Carmichael James, Ward Wynne, Hammonds Timothy R. and Stevens Malcolm F.G. Antitumor Polycyclic Acridines. 7. Synthesis and Biological Properties of DNA Affinic Tetra- and Pentacyclic Acridines . *J. Med. Chem*. **2000**, 43, 8, 1563–1572 https://doi.org/10.1021/jm9909490
2. Hagan Damien J., Giménez-Arnau Elena, Schwalbe Carl H. and Stevens Malcolm F. G. Antitumour polycyclic acridines. Part 1. Synthesis of 7H-pyrido- and 8H-quino-[4,3,2-kl]acridines by Graebe–Ullmann thermolysis of 9-(1,2,3-triazol-1-yl)acridines: application of differential scanning calorimetry to predict optimum cyclisation conditions. *J. Chem. Soc*., Perkin Trans. 1, **1997**, 2739-2746 https://doi.org/10.1039/A702299I
3. Hutchinson Ian, McCarroll Andrew J, Heald Robert A. and Stevens Malcolm F. G. Synthesis and properties of bioactive 2- and 3-amino-8-methyl-8H-quino[4,3,2-kl]acridine and 8,13-dimethyl-8H-quino[4,3,2-kl] acridinium salts. *Org. Biomol. Chem*., **2004**, 2, 220-228 https://doi.org/10.1039/B310796P
