# Supplementary material for: Effects of the Combined Treatment with a G-Quadruplex-Stabilizing Ligand and Photon Beams on Glioblastoma Stem-like Cells: A Magnetic Resonance Study
Source: Int J Mol Sci. 2021 Nov 24;22(23):12709. doi: 10.3390/ijms222312709 (PMC8657890; doi:10.3390/ijms222312709)
Supplement: Supplementary file 1 [file ijms-22-12709-s001.zip › Supplementary Table S1.pdf]

**Supplementary Table S1. Patient demographics, clinical information and GBM features.**

| | Age | Sex | Previous chemo/RT | Up-front/ recurrent | IDH | Core/periphery |
|---|---|---|---|---|---|---|
| **GSC #1** | 40 | M | no | Up-front | wild type | Core |
| **GSC #83** | 52 | M | no | Up-front | wild type | Core |
